# Deep Bleeder Acoustic Coagulation (DBAC)—part II: in vivo testing of a research prototype system

**DOI:** 10.1186/s40349-015-0038-3

**Published:** 2015-09-24

**Authors:** K. Michael Sekins, Stephen R. Barnes, Liexiang Fan, Jerry D. Hopple, Stephen J. Hsu, John Kook, Chi-Yin Lee, Caroline Maleke, Xiaozheng (Jenny) Zeng, Romain Moreau-Gobard, Alexis Ahiekpor-Dravi, Gareth Funka-Lea, John Eaton, Keith Wong, Scott Keneman, Stuart B. Mitchell, Barbrina Dunmire, John C. Kucewicz, Fred J. Clubb, Matthew W. Miller, Lawrence A. Crum

**Affiliations:** Siemens Ultrasound Business Unit, 22010 S.E. 51st Street, Issaquah, WA 98029-1271 USA; Siemens Corporate Research and Technology, 755 College Road East, Princeton, NJ 08540 USA; Center for Industrial and Medical Ultrasound, Applied Physics Laboratory, University of Washington, 013 NE 40th Street, Seattle, WA 98105-6698 USA; ETN LLC, 1150 Guinda St., Palo Alto, CA 94301 USA; Texas Institute for Preclinical Studies (TIPS), Texas A&M University, College Station, TX 77843 USA; Department of Veterinary Pathology, Texas A&M University, 4467 Veterinary Medical Science Building, College Station, TX 77843 USA; 8808 Points Dr. N.E., Yarrow Point, WA 98004 USA

## Abstract

**Background:**

Deep Bleeder Acoustic Coagulation (DBAC) is an ultrasound image-guided high-intensity focused ultrasound (HIFU) method proposed to automatically detect and localize (D&L) and treat deep, bleeding, combat wounds in the limbs of soldiers. A prototype DBAC system consisting of an applicator and control unit was developed for testing on animals. To enhance control, and thus safety, of the ultimate human DBAC autonomous product system, a thermal coagulation strategy that minimized cavitation, boiling, and non-linear behaviors was used.

**Material and methods:**

The *in vivo* DBAC applicator design had four therapy tiles (Tx) and two 3D (volume) imaging probes (Ix) and was configured to be compatible with a porcine limb bleeder model developed in this research. The DBAC applicator was evaluated under quantitative test conditions (e.g., bleeder depths, flow rates, treatment time limits, and dose exposure time limits) in an *in vivo* study (final exam) comprising 12 bleeder treatments in three swine. To quantify blood flow rates, the “bleeder” targets were intact arterial branches, i.e., the superficial femoral artery (SFA) and a deep femoral artery (DFA). D&L identified, characterized, and targeted bleeders. The therapy sequence selected Tx arrays and determined the acoustic power and Tx beam steering, focus, and scan patterns. The user interface commands consisted of two buttons: “Start D&L” and “Start Therapy.” Targeting accuracy was assessed by necropsy and histologic exams and efficacy (vessel coagulative occlusion) by angiography and histology.

**Results:**

The D&L process (Part I article, J Ther Ultrasound, 2015 (this issue)) executed fully in all cases in under 5 min and targeting evaluation showed 11 of 12 thermal lesions centered on the correct vessel subsection, with minimal damage to adjacent structures. The automated therapy sequence also executed properly, with select manual steps. Because the dose exposure time limit (*t*_dose_ ≤ 30 s) was associated with nonefficacious treatment, 60-s dosing and dual-dosing was also pursued. Thrombogenic evidence (blood clotting) and collagen denaturation (vessel shrinkage) were found in necropsy and histologically in all targeted SFAs. Acute SFA reductions in blood flow (20–30 %) were achieved in one subject, and one partial and one complete vessel occlusion were confirmed angiographically. The complete occlusion case was achieved with a dual dose (90 s total exposure) with focal intensity ≈500 W/cm^2^ (spatial average, temporal average).

**Conclusions:**

While not meeting all in vivo objectives, the overall performance of the DBAC applicator was positive. In particular, D&L automation workflow was verified during each of the tests, with processing times well under specified (10 min) limits, and all bleeder branches were detected and localized. Further, gross necropsy and tissue examination confirmed that the HIFU thermal lesions were coincident with the target vessel locations in over 90 % of the multi-array dosing treatments. The SFA/DFA bleeder models selected, and the protocols used, were the most suitable practical model options for the given DBAC anatomical and bleeder requirements. The animal models were imperfect in some challenging aspects, including requiring tissue-mimicking material (TMM) standoffs to achieve deep target depths, thereby introducing device-tissue motion, with resultant imaging artifacts. The model “bleeders” involved intact vessels, which are subject to less efficient heating and coagulation cascade behaviors than true puncture injuries.

## Background

### Deep Bleeder Acoustic Coagulation (DBAC)

A Deep Bleeder Acoustic Coagulation (DBAC) cuff system is an ultrasound device intended to cauterize deep, bleeding, combat limb wounds of soldiers while in the field. This paper is a companion to the Part I article [1] that describes the development and *in vitro* (phantom) testing of a DBAC cuff research prototype. The DBAC system is designed to operate autonomously, first to detect and localize (D&L) deep arterial bleeders with ultrasound imaging and treat them using high-intensity focused ultrasound (HIFU) [1]. The primary objective of the project described herein was the demonstration of acoustic coagulation using an automated DBAC device in an animal model over a range of target depths, bleeder flow rates, and treatment time limits as defined in the project contract.^1^ The device tests were administered in an in vivo test bed final exam series of evaluations developed and performed in collaboration with two laboratories. A high-level outline of the required test and parameter-range conditions is shown in Table 1.

**Table 1:** Outline of test conditions/objectives for in vivo DBAC testing

	Milestone	Most challenging problem	Milestone specification	Treatment speed	Device operation
DBAC hemostasis test condition/objective	Slow bleeder injury	Slow bleed in small vessel	5 ml/min	Achieve hemostasis before 50 % blood loss. Maximum time limit of 30 min	Full automation: start button, cancel button Closed-loop system Minimum of two panels required
	Fast bleeder injury	Fast bleed in large vessel	100 ml/min		
	Depth of penetration	Large, fast, deep bleeder	12.5 cm		
	Maximum thermal tissue dose	Large, fast, deep bleeder	Thermal lesioning limited to 2-cm sphere centered on the bleed		
	Maximum thermal skin dose	Skin burn	MTSD ≤ 52 °C		

### DBAC animal model

In vivo test conditions differed from those for the in vitro case [1], in part because bleeder flow rates and depths in an animal cannot be as precisely prescribed as in a phantom due to physiologic variability and the effects of invasive instrumentation. For in vivo DBAC testing to occur, animal model development was therefore required, with a variety of candidate animal models being evaluated, both analytically and through in vivo experiments. The animal selected was to preferably have human adult limb-like “cuff compatible” anatomy, and the model was to enable quantitative control of bleeder blood flow and vascular parameters. These included maintaining suitable normal and bleeder arterial flow waveforms and providing an in vivo “tourniquet function,” whereby the bleeding could be stopped during the HIFU treatment, as explained in reference [1].

Model development involved evaluating ovine, canine, and porcine species, focusing on experimentally evaluating both cervical (carotid artery) bleeders and femoral artery bleeders. Integral to the final model selection was the conclusion that a full (360°) cuff DBAC device, such as developed and described in [1], could not be tested in a practical animal model and hence required some compromise.

**Fig. 1:**
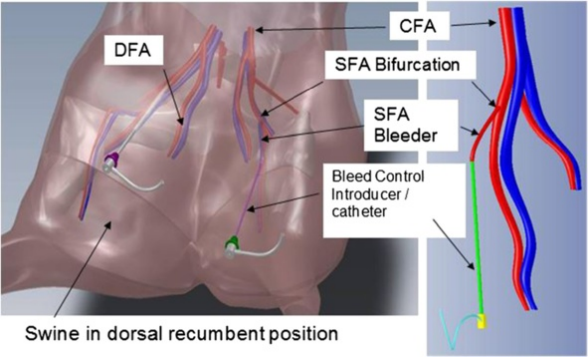
DBAC in vivo bleeder model: porcine superficial femoral artery (SFA). CAD drawing from CT scans and angiograms of vascular anatomy. The sheath was inserted into the SFA, and depending on the test subject, the bleeder was defined to be 10 mm or 20–25 mm distal to the SFA bifurcation with the common femoral artery (CFA)

The compromise was to test a DBAC cuff “subsection” on the hind limbs of large swine, using the intact (not punctured) superficial femoral artery (SFA) as the simulated bleeder and “bleeder track” (Fig. 1). While an actual arterial puncture may have more closely represented arterial bleeding, this approach would not have allowed for practical monitoring and control of bleeding flow rates, and it would not easily enable the needed “tourniquet function.” A continuation segment of the femoral artery beyond the SFA bifurcation, referred to here as the deep femoral artery (DFA), was also used as a bleeder in some animals to access the deepest bleeder targets.

## Material and methods

### In vivo animal DBAC device

**Fig. 2:**
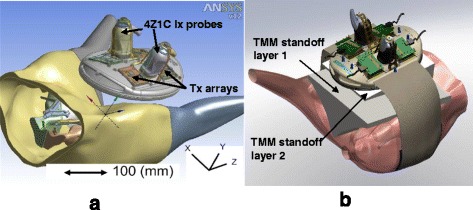
Experimental configuration during DBAC animal testing. Shown are CAD model depictions of the following: **a** the in vivo DBAC applicator with the 3D imaging probes (4Z1c) on porcine groin targeting SFA bleeder; **b** in vivo DBAC device used with TMM tissue standoffs targeting bleeders and different depths

Several design iterations of the in vivo DBAC prototype device were required to adequately modify the in vitro cuff geometry into a subsection of the prototype “human cuff” suitable for the intended testing. The design, optimized using computational modeling and the animal model development tests, comprised an elliptically shaped rigid plastic support structure incorporating a sealed, water-filled bolus for acoustic coupling, with the positions of the 2D therapy arrays (the HIFU “tiles” or “Tx”) and volume imaging arrays (“Ix”) being identical to a contiguous section of the full cuff prototype described in [1]. The power and control unit was architecturally (acoustically, electronically, and in software) very similar to that used in the in vitro DBAC research prototype. Figure 2a shows the in vivo applicator configuration superposed on the porcine groin. To achieve the required bleeder depth ranges (3.5 to 12.5 cm) using the shallow SFA (≈2-cm deep), an added “tissue layer” was placed between the skin surface of the animal and the DBAC applicator. Figure 2b depicts this arrangement in which acoustically and thermally appropriate tissue-mimicking material (TMM) standoff layers of different thicknesses (depending on desired bleeder depth) were placed on the porcine groin. To provide a flexible and conformal standoff, a 10 % polyvinyl alcohol (PVA)-based TMM with aluminum oxide attenuating scatterers was used [2]. Up to two TMM layers of different thicknesses were used to achieve the deepest required bleeder target depths.

The applicator’s cuff-like curvature was implemented by having two surfaces set at a 30° angle between them, whereby each surface substituted for a cuff “panel” of the research prototype, each having two Tx’s and one Ix. Imaging was accomplished with Siemens 4Z1c probes (acoustic center frequency, *f*_c_ = 2.5 MHz) connected to a Siemens ACUSON SC2000™ ultrasound imaging system [1]. The in vivo applicator enabled testing of key DBAC cuff functionality on the porcine SFA model, which had arteries of an appropriate physical scale and allowed vascular access for blood flow quantitative control, including a tourniquet mechanism.

**Fig. 3:**
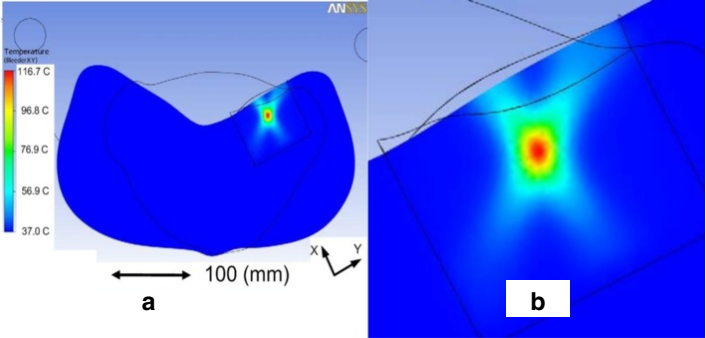
Example of simulated cross-sectional temperature distributions. To guide applicator positioning and TMM standoff requirements, **a** 3D acousto-thermal FEM models were used incorporating a perfused anterior limb muscle field near the SFA. **b** Inset showing a *T*_eod_ distribution

**Fig. 4:**
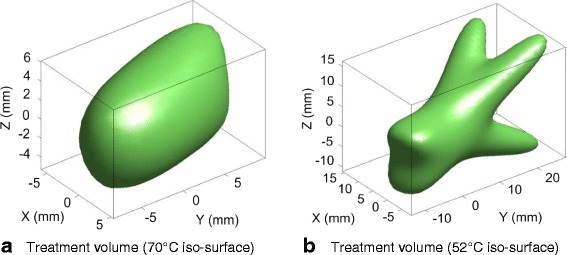
Sample simulated thermal treatment volumes produced by four Tx tiles of the in vivo DBAC applicator. Enclosed end-of-dose tissue volumes are at and above the iso-surface temperature indicated.  **a** indicates the end-of-dose inner core (target tissue) volume over a therapeutic temperature threshold of 70 °C; **b** shows the larger region surrounding the core target region that exceed 52 °C (all dimensions are in mm)

To estimate individual Tx powers and beam patterns during dosing, and to provide device positioning guidance, the treatment tissue thermal responses were computationally simulated in finite element method (FEM) models (ANSYS v.12 and ANSYS Multi-Physics codes; ANSYS Inc., Canonsburg, PA, USA). The model was based on the anatomy and coordinates of the SFA bleeders and the geometry of the Tx tiles, extracted from applicator-on-animal computer-assisted design (CAD) models (SolidWorks, Dassault Systemes Solidworks Corp., Waltham, MA, USA), as in Fig. 2. The acoustic fields were input as distributed energy sources into the FEM tissue models, which incorporated the bio-heat transfer equation [3] to take into account perfusion influences on temperatures. Sample end-of-dose temperature (*T*_eod_) distributions are shown in Fig. 3, with detailed focal heating volumes in Fig. 4 (generated according to the geometry of Fig. 2). Simulations of the thermal response of the target vessel (e.g., SFA blood flow convective effects) are discussed below.

In the Fig. 4 example, the time-averaged acoustic power for each Tx was ≈50 W with a dosing time of 60 s. The 70 °C iso-surface-enclosed volume in Fig. 4a approximates the smallest typical lesion. Since tiles on the in vivo applicator did not have as wide an angular distribution of acoustic power as a cuff, the resulting heated region shape (see Fig. 4b), while having a circular lateral cross-section, is long in the axial direction.

### In vivo test bed protocol

The animal model and test protocols were developed using a total of 45 swine, with the in vivo final exam and its finalization utilizing an additional five animals.^2^ Immediately prior to the final exam, practice tests on two swine were used to finalize the protocol and verify the compatibility and suitability of the test measurement methods. In the final exam, performed in accordance with approved Texas A&M Institute for Preclinical Studies (TIPS) Animal Use Protocols [4], 12 bleeder treatments were performed in three animals.

**Fig. 5:**
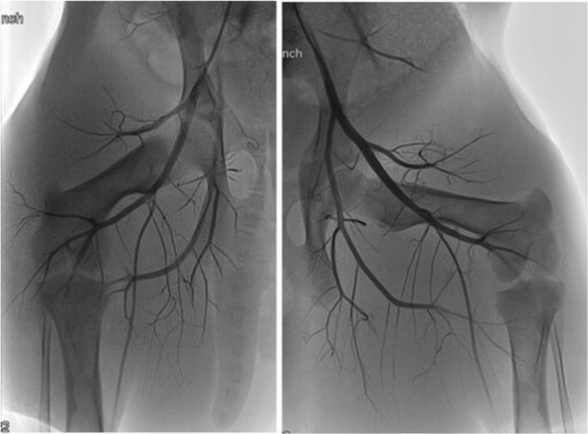
Pre-treatment angiograms of both the right and left rear limbs. In all tests, these steps were taken to characterize the vascular anatomy prior to instrumentation and to provide baseline images to which the post-therapy angiograms could be compared

Animals were anesthetized, and the caudal ventral abdomen and inner thighs were prepared using a cream depilatory to remove all hair. Systemic heparinization was used due to the invasive instrumentation, and baseline ultrasound gray scale imaging (B-mode), color-flow Doppler, and spectral Doppler (SD) ultrasound scans were performed to characterize the vascular anatomy. Baseline fluoroscopy (Fig. 5) was also performed, with both right and left common carotid arteries being surgically exposed and 8- to 10-French catheter introducers positioned in each. A multi-side-hole diagnostic catheter (for dye injection) was advanced under fluoroscopic guidance to the distal aorta, and angiography of the hind limb vasculature was performed pre-treatment.

**Fig. 6:**
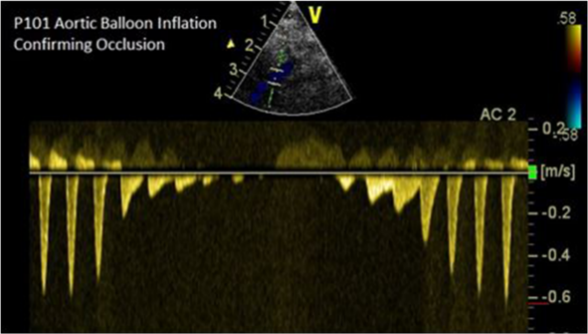
Spectral Doppler (SD) flow confirmation of “tourniquet” function of the intra-aortic balloon. Flow in the femoral artery was eliminated by balloon inflation and returned rapidly with balloon deflation. SD evaluation was repeated following therapy

For blood flow control during treatment, balloon-tipped catheters (with balloons 16- to 18-mm diameter; 4-cm length) were advanced alongside the aforementioned diagnostic catheter to an aortic position distal to the renal arteries but proximal to the external iliac arteries. The balloon catheter was inflated and, using the previously positioned diagnostic catheter, an aortogram was performed to confirm appropriate positioning of the balloon catheter, as well as to enable complete occlusion of the distal aorta. This catheter access, along with stopcock manipulation in the SFA-resident catheter, was used to alter flow in the common femoral and SFA arteries. This allowed fine adjustment of volumetric bleed rates and complete occlusion of femoral arterial flow (the “tourniquet” function) during therapy. B-mode and Doppler flow scans (Fig. 6) were repeated with the balloon catheter first deflated, then gradually inflated to confirm that flow could be decreased, and ultimately stopped, based on the degree of balloon inflation. This process was also repeated immediately prior to euthanasia to confirm the reliability of the “tourniquet” function of the aortic balloon.

For animal stability, the “tourniquet shut-off” could not be maintained throughout the therapy periods in some “extended dose” treatments, since the fully inflated balloon catheter cut off perfusion to almost half of the animal. To expose the distal SFA for placement of the introducer/sheath, a small surgical cut-down was performed and a 4-French introducer/sheath advanced approximately 2–4 cm into the bleeder arterial lumen (Fig. 1).

Using this setup, a series of in vivo controlled bleed studies were performed using the balloon catheter degree-of-inflation and introducer catheter stopcock settings. The applicator was also evaluated for compatibility with the animal preparation. “Bleeder” flow waveforms were created by altering inflow into the common femoral artery (CFA) using the balloon-tipped catheters and allowing the catheter introduced into the SFA to vent its flow to the atmosphere. During “bleeder tract” characterization by the DBAC system, a bleeder waveform was defined by a low Resistive Index (RI<0.75) [1]. Volumetric flow rates were confirmed by collecting shed blood over a measured time period into a clean bowl and then transferring that blood into a validated graduated cylinder. Importantly, once shed blood volume was measured, it was returned to the study subject to preserve the hemodynamic stability of the model. This process was repeated during the testing sessions to ensure accurate volumetric bleed data.

It was found in some animals after the SFA catheterization had been completed that the bleeder target did not meet the deepest requirements, even with the thickest (6 cm) standoff. It was in these situations that the deep femoral artery (DFA) was selected as the bleeder target. Regrettably, the DFAs under these circumstances could not be instrumented with introducer catheters (Fig. 1).

Regarding test conditions, the control of depth and blood flow rate were categorized into coarse ranges, as follows:

Bleeder rate and depth range test condition objectives:

**Table:** 

Slow bleed	Blood flow rate = 5 to 60 ml/min
Fast bleed	Blood flow rate = 60 to 100+ ml/min
Shallow depth	Bleeder depth = 3.75 to 8 cm
Deep depth	Bleeder depth = 8 to 12.5 cm

The efficacy objective was to produce full obstruction of the lumen of the SFA via thermal coagulation mechanisms induced by confocal beams from the in vivo DBAC applicator. The SFA bleeder model had the advantage of the blood not being prone to spontaneous coagulation (no device/blood contact in the region), and the bleeder target region of interest was free of stitches, vascular clamps, or other hardware.

Following completion of an in vivo study, angiograms were repeated, and the adequacy of anticoagulation was again confirmed before the study subjects were euthanized. Complete postmortem examinations of the vascular regions of interest were performed, including lesions being photographically documented and samples being obtained for histologic evaluation.

**Fig. 7:**
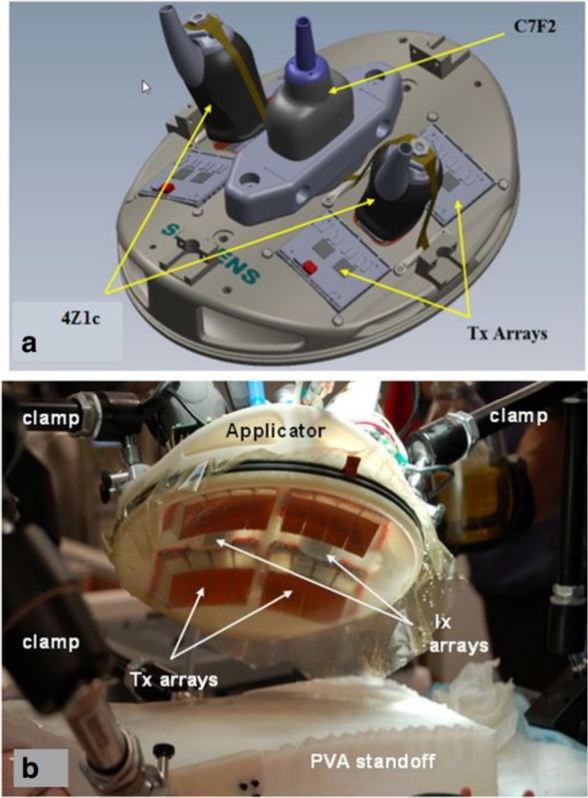
In vivo DBAC device and final exam treatment setup. **a** Applicator showing four therapeutic array tiles, the two 4Z1c 3D imaging probes, and backup 2D C7F2 imaging probe; **b** applicator being positioned for treatment on PVA TMM standoff (secured by Manfrotto™ clamps attached to surgical table)

The TMM standoffs were ultimately determined to have a non-negligible impact on acoustic performance (for both imaging and therapy). For this reason, it was useful to deploy a backup imaging probe (in addition to the 4Z1c) to corroborate and supplement the 4Z1c, particularly for the deepest bleeders. Accordingly, although not part of the DBAC applicator design, a 2D imaging probe with more penetration (Siemens C7F2) was installed on the applicator (Fig. 7a). The applicator had a closed water-filled coupling compartment surrounding the transducers, created via a 50-μm polyurethane membrane stretched across the bottom opening of the applicator and secured with two O rings (Fig. 7a, b). Fill and vent ports were used for priming the coupling compartment with degassed water, which also enabled air to be purged. As appropriate, the applicator was either positioned on the subject’s skin surface or the TMM standoff, with acoustic gel or lotion used to couple the interfaces (i.e., membrane TMM and TMM animal skin). The in vivo applicator and the TMM standoff layers were held in place on the animal with clamps (Fig. 7a, b). TMM-associated imaging noise occurred primarily at the TMM-TMM and TMM-skin interfaces, and this noise was addressed in part by the use of custom image processing algorithms designed to filter and suppress noise at the interfaces.

### Treatment workflow and automation

**Fig. 8:**
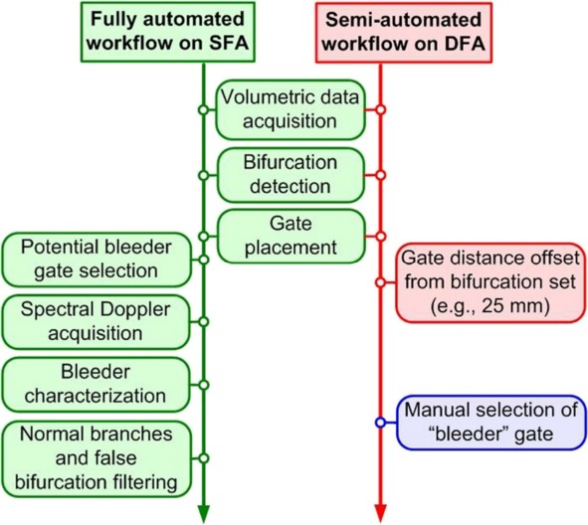
Detection and localization (D&L) workflows

**Fig. 9:**
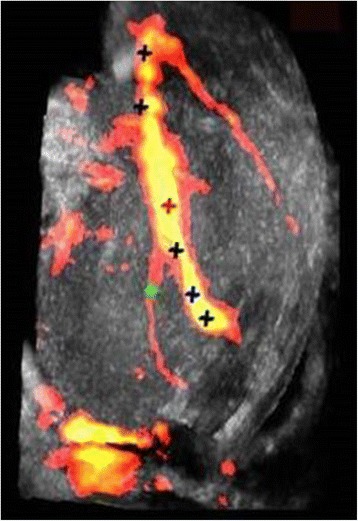
Image of limb vascularity in a test subject with SFA bleeder branch. Compound 3D power Doppler image with D&L automated algorithm-placed markers (*crosses*) denoting normal branches (bifurcations), vascular centerline markers, and the bleeder target (*green cross*), here 10 mm distal to the bleeder SFA branching point

Similar to the in vitro DBAC treatment [1], an objective was a “two-button” treatment procedure available to the operator: Start D&L and Start Therapy. As depicted in Fig. 8, D&L included (a) B-mode and power Doppler mode acquisitions, (b) bleeder detection, (c) bleeder (or normal branch) characterization via resistive index, and therapy marker placement on the vessel target. Figure 9 shows a sample power Doppler image used for vascular branch characterization and bleeder (SFA branch) localization. If a DFA vessel segment was the designated target, the therapy marker was placed manually (since the DFA was not catheterized). The communication between D&L and therapy subsystems was also established at the end of the D&L sequence.

Upon D&L sequence completion, an automatic pause state was entered, to allow synchronization with the animal preparation workflow involving the arterial catheter balloon inflation. After inflation of the balloon catheter, simulating tourniquet deployment, the “Start Therapy” button was launched, which completed the treatment cycle. Tx array driver voltages, whose values were determined automatically, were manually set on the DBAC power supply. After the target location was passed from D&L processing to the therapy subsystem automatically, the dose was calculated, the recruited therapy tiles were selected, and the duty cycles for each individual Tx were determined, all automatically. The targeting and therapy mode states on the ACUSON SC2000 system were also automatically set.

Of the 12 bleeder treatments, the DFA was used in 6 and could not exhibit “bleeder” pulsatility waveforms since no venting (via catheterization) was available. For the “DFA bleeder,” a manual (non-automated) therapy marker was thus used to target the bleeder. While the SFA workflow was fully automated, the DFA had this manual step (Fig. 8).

### Targeting and dosing

DBAC closed-loop targeting, based on thermal strain imaging of the focus, and closed-loop dosing, based on recurrent neural network (RNN) acoustic thermometry, were demonstrated earlier to be reasonably effective for the in vitro cuff prototype testing in phantoms [1]. However, only a few animal experiments incorporating targeting and thermometry data acquisition were performed prior to the final exam. Thus, available data was insufficient for “training” and validating the in vivo acoustic radiation force impulse (ARFI) imaging and RNN thermometry algorithms, particularly with in vivo tissue motion. Therefore, while automated closed-loop targeting and dosing was not used formally to guide treatments, RNN thermometry (based on ex vivo liver data) was used to visually assess the locations of energy delivery relative to the desired targets and to estimate dosing relative temperatures. The in vivo automated targeting was instead achieved by D&L alone, without iterative targeting correction as described in [1]. In this processing, the bleeder location was converted to coordinates in “therapy space,” and dosing power levels and beam patterns were then auto-computed to produce coagulative necrosis at that location under fixed dose exposure time conditions.

The objective that *T*_eod_ at the skin surface not exceed 52 °C under worst case dosing conditions was tested as follows: (a) a deep target was selected in a single animal (away from the SFA and DFAs being treated); (b) a 75-μm butt-welded thermocouple was placed at the interface between the PVA (virtual “skin”) and the in vivo applicator fluid compartment skin-contacting membrane; (c) using the geometry of the tiles and the target for guidance, the thermocouple junction was positioned within the cross-section of the least steered therapy beam at the interface; and (d) a maximum power dose at 60-s exposure was delivered and the resulting thermocouple temperature rise recorded.

## Results

### Detection and localization (D&L)

D&L assessment focused on the following main measures: automated D&L workflow success, detection of both slow and fast bleed injuries, achieving and detecting the deepest bleeds, and keeping combined D&L plus therapy time ≤30 min.

**Fig. 10:**
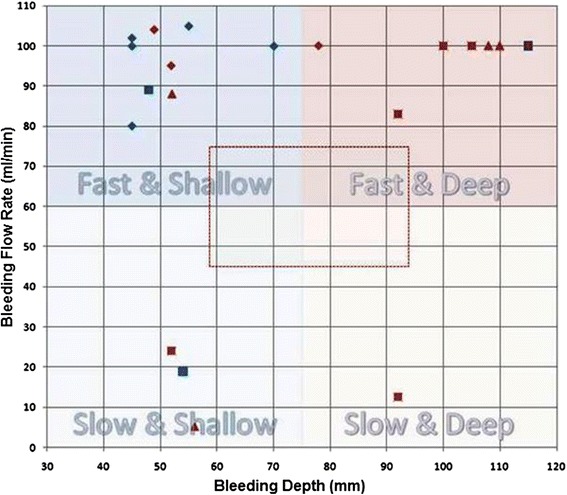
D&L bleeder test cases in the in vivo test bed. General test range area avoided is depicted with the *red-box outline*. Legend: *blue diamond symbol* = practice animal 1; *blue square symbol* = practice animal 2; *red diamond symbol* = test animal 1; *red square symbol* = test animal 2; *triangle red symbol* = test animal 3

Figure 10 and Table 2 summarizes the D&L test conditions and bleeder characterization success rates. Overall, D&L results were positive with all bleeder branches being detected and localized and with one-button D&L automation workflow verified during each of the tests. Image acquisition plus D&L processing times were less than 5 min for the first bleeder, well under the limit of 10 min. The 4Z1c-probe-based D&L times for the SFA averaged 265 s for first bleeders, and 25 s for subsequent bleeders. The C7F2 probe was used for DFA-vessel D&L (again, manually assigning location markers) and had an average D&L time of 203 s. Two specific bleeder characterizations were problematic in being incorrectly characterized as non-bleeders; both were slow bleeders (24 and 5 ml/min cases).

**Table 2:** Summary of in vivo final exam D&L results

Exp. no.	Side	Bleed type	Bleed depth (mm)	Depth category	Flow rate (ml/min)	Flow rate category	Bleed character correct?
1	L	SFA	49	Shallow	104	Fast	Y
2	L	DFA	115	Deep	N/A		
3	R	SFA	52	Shallow	95	Fast	Y
4	R	DFA	78	Deep	N/A		
5	R	SFA	52	Shallow	24	Slow	Y
6	R	SFA	52	Shallow	24	Slow	N^a^
7	R	DFA	105	Deep	N/A		
8	L	SFA	92	Deep	12.5	Slow	Y
9	L	SFA	92	Deep	83	Fast	Y
10	L	DFA	110	Deep	N/A		
11	R	DFA	110	Deep	N/A		
12	R	SFA	56	Shallow	5.2	Slow	Y
13	R	SFA	56	Shallow	5.2	Slow	N^a^
14	L	DFA	108	Deep	N/A		
15	L	SFA	52	Shallow	88	Fast	Y

Exps 1–4: animal #101; Exps 5–10: animal #104; Exps 11–15: animal #103

^a^Bleeder incorrectly characterized by algorithm as non-bleeder

### Targeting and dosing

**Fig. 11:**
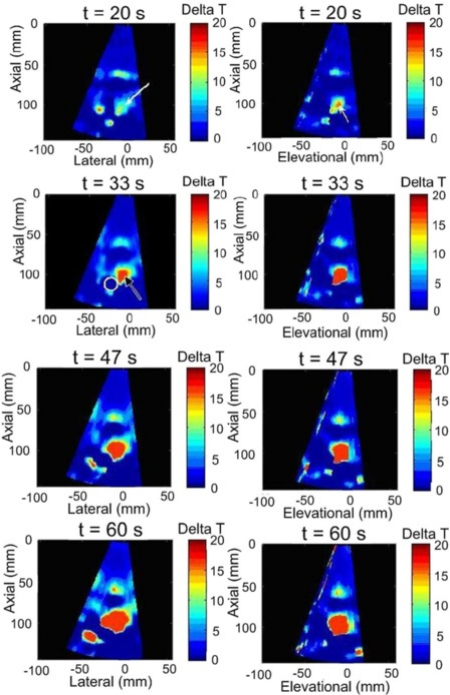
Target temperature evolution during dosing in in vivo porcine muscle. Target is DFA, left hind limb. Left column: axial-lateral plane views. Right column: axial-elevational views. Color bar: ∆*T* = 0 °C to 20 °C (note: RNN thermometry was based on algorithm training from ex vivo liver data). In the first three figures the beam direction and focus are marked with *white* and *black arrows*, respectively and the cross-section of the vessel is annotated with a *white circle*

Figure 11 displays a time series of RNN temperature maps from a single animal treatment to illustrate the use of RNN thermometry to ascertain whether acoustic energy was delivered to desired target locations. The SFA and DFA coagulation results are shown in Table 3.

**Table 3:** Summary of in vivo test bed final exam therapy results

Test no.	Vessel type	Depth range	Depth of penetration	Dosing time (s)		Input acoustic power (W)	Focal intensity^a^ (W/cm^2^)	Lesion^b^	Vessel occlusion^c^
1	SFA	Shallow	4.7	30	30^e^	225	472	Y	N
2	DFA	Deep	11.5	30		378	472	Y	N
3	SFA	Shallow	5.5	30	30	472	472	N	N
4	DFA	Shallow^d^	7.8	30	30	472	472	Y	N
5^g^	SFA	Shallow	5.2	60		257	472	Y	Partial
6	DFA	Deep	10.5	60		211	266	Y	N
7	SFA	Deep	9.2	60		195	266	Y	N
8	DFA	Deep	11.0	60		218	259	Y	N
9	DFA	Deep	10.8	18	60	225	266	Y	N
10^h^	SFA	Shallow	5.2	60	30^f^	257	472	Y	Y
11	DFA	Deep	10.7	60		223	266	Y	N
12	SFA	Shallow	5.6	60		249	486	Y	N

Exps 1–4: animal #101; Exps 5–8: animal #104; Exps 9–12: animal #103

^a^Intensity values are spatial average and temporal average; all foci superposed with attenuation derated according to each respective beam path

^b^Status as regards the question: lesion around target region? (targeting assessment)

^c^Occlusion determined by reduction or stop of flow as evidenced in post-therapy angiography

^d^Although classified as shallow, location was at the border of the shallow-deep transition depth

^e^Prolonged dosing procedure: second dose delivered 27 min after first dose; also one Tx (25 % of aperture) hit array thermal shutoff limits during 30-s dosing

^f^Prolonged dosing procedure: second dose delivered 37 min after first dose

^g^Partial occlusion animal (#104); skin burns noted with TMM divoting of the PVA

^h^Complete occlusion animal (#103)

**Fig. 12:**
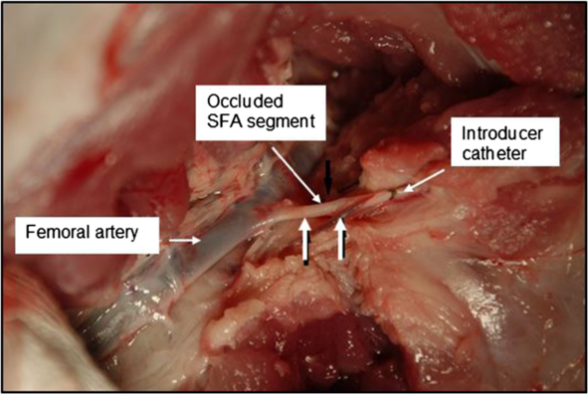
Immediate postmortem evaluation of the SFA in animal #103. (Tissue exam from that of test #10 in Table 3.) Note the dramatic difference in the visual appearance of the common femoral artery and the SFA target vessel (*vertical arrows*)

The accuracy of targeting (“Lesion” column in Table 3) was by gross necropsy evaluation, with histopathology of border samples from the lesions produced. Immediate postmortem tissue examinations were performed to assess the location (in comparison with intended target locations) and extent of the thermal lesioning in the vessel target areas. These visual inspections confirmed that the lesions were almost exclusively coincident with the distal branch locations targeted (Fig. 12). Gross necropsy confirmed the presence of lesions in appropriate anatomic locations in 11 of the 12 (91.6 %) targets, as indicated in Table 3. Measurement or calculation of entire lesion volumes could not be performed accurately while still allowing dissection and documentation of lesions. Lesion diameter was measured, however, and ranged between 7 and 19 mm, with most approximately 11 mm.

### Coagulation endpoints

Although the maximal focal intensities for individual (static) HIFU beams were ≈1600 W/cm^2^, the intensities reported in Table 3 reflect values that were spatially averaged over the scanned multi-foci pattern (an 8-mm diameter “dithering” pattern, as discussed in [1]) then derated for attenuation along the beam path for all Tx beams. The intensities are the result of superposition of all the Tx beam foci.

**Fig. 13:**
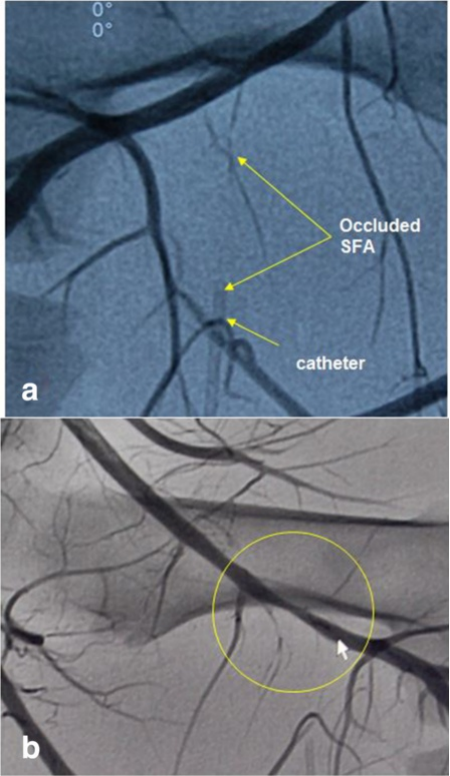
**a** Complete bleeder track occlusion. Angiogram in animal #103 (test #10 in Table 3) showing (post-treatment) the occluded SFA tract. **b** Partial bleeder track occlusion. Angiogram showing significantly narrowed femoral artery at the branch point of the SFA in animal #104 (test #5 in Table 3). A subsequent angiogram documented substantially reduced flow through the SFA. Histology showed notable changes to the CFA in this treatment, unlike the “parent vessels” in all other treatments

The results of the two practice-animal tests revealed a pattern of not achieving definitive efficacy (hemostasis; defined as arterial occlusion) using the required ≤30-s dose regimes. Accordingly, dose escalation, including applying two doses, was used in subsequent animals, achieved primarily by extending the total cumulative high power exposure times beyond 30 s (Table 3). Of significance in the cases of dual dosing, tissue cooldown to baseline occurred between doses due to the extended times (5 to 37 min) needed to measure blood flow rates after the first dose (involving balloon catheter deflation, hemodynamic stabilization, and re-inflation). Regarding efficacy, acute reductions in blood flow (20 and 30 %; measured 10 min post-dose) were found for two SFA targets in animal #101. One partial vessel occlusion (Fig. 13b) and one complete occlusion (Fig. 13a) were confirmed angiographically.

**Fig. 14:**
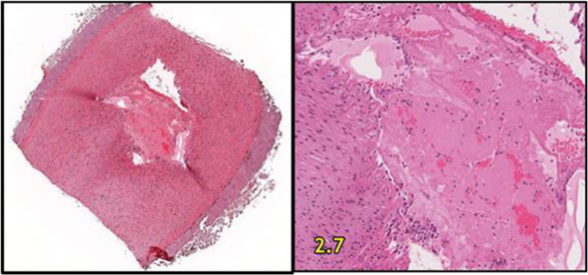
Histopathologic evaluation of the right SFA of animal #103. (Tissue exam from that of Test #10 in Table 3.) Demonstrates complete luminal occlusion (*left panel*). Additional findings included transmural necrosis of smooth muscle cells in the vessel media and marked basophilic degeneration of the collagenous substrate in the adventitial region

Histopathology also showed increased blood clotting (thrombogenesis) for the latter tests as acoustic dose exposures were increased. Figure 14 shows the histologic changes in the target vessel of animal #103 (Figs. 12 and 13a), in which the right femoral artery became completely occluded (post-dosing) by thermally induced thrombus. Particles of thrombus material were noted and removed from the lumen during examination. The degree of change in the SFA branch in this treatment was quite evident during gross postmortem examination as well. Despite complete occlusion of the SFA in this case, the cross-section of the parent vessel (the CFA at the branching point) was histologically normal, supporting localization and targeting accuracy.

**Fig. 15:**
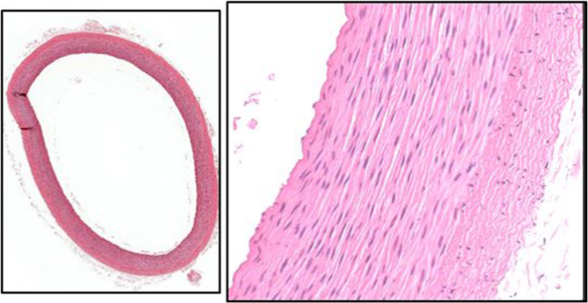
Representative histologic section from parent vessel immediately proximal or distal to target vessels. The lack of histologic changes in the vessels in intimate proximity to the target area is believed to support the accuracy of the targeting

Targeting and therapeutic dosing for almost all cases were sufficiently well controlled to yield minimal collateral damage. Lesions created on the SFA, the primary bleed target site, were consistently confined to the target area with minimal or no changes noted in the parent vessel (Fig. 15). The notable exception was animal #104 (test #5), showing partial occlusion, in which the parent vessel showed evidence of modest histologic changes, as well, and mild angiographic changes. In this case, the target vessel had the most dramatic histologic changes of the bleeder vessels treated in the series. Microscopic evaluation of this treated SFA area showed loss of endothelial lining, and approximately 80 to 90 % of the cross-sections had acute necrosis (characterized by pyknotic loss of nuclei) and hyper-eosinophilia (with effaced architecture) of smooth muscle cell cytoplasm. Additionally, the lumen of the vasa vasorum, located in the adventitia, was distended by the blood coagulum.

The objective of maximum skin temperature, *T*_eod_ ≤ 52 °C, under worst case dosing conditions was met. A target was selected at 10.8 cm depth (beneath the 6 cm PVA standoff), and a 60 s dose at maximum power, 211 W_ac_, was delivered. As measured by the thermocouple, the resulting surrogate skin (TMM surface) Δ*T*_eod_ ≈ 2.6 °C, equivalent to a maximum *T*_eod_ = 39.6 °C.

## Discussion

### Detection and localization

All in vivo D&L depth and flow test conditions were tested (noting that the deepest bleeder was 11.5 cm depth; see below), and cumulative acquisition and D&L time were kept under 5 min, well below the required 10-min limit*.* The lack of closed-loop targeting (depending only on initially determined D&L coordinates) was not significantly limiting since, in almost all cases, post-treatment necropsy showed thermal lesioning approximately coincident with the SFA or DFA branches at the target locations. Overall, D&L results were positive (92 % success rate based on postmortem tissue exams). It is understood that the importance of feedback-controlled targeting would be greater in the case of a true DBAC cuff, given that multiple beams must be confocal from a surrounding 360° acoustic aperture.

### Animal model

In investigating alternatives to obtain a physically deeper target than the SFA, a complementary bleeder model preparation involving the profunda femoral artery branch was experimentally evaluated. Although potentially providing deeper bleeder targets than the SFA, the profunda was found difficult to instrument and perform the post-therapy cut-down necropsy. Thus, the DFA was determined the best alternate to the SFA.

The TMM standoffs enabled treatment of deeper bleeder targets but requiring certain tradeoffs in test logistics and acoustic performance. Although using multiple standoffs to achieve the maximum depth of 12.5 cm was attempted, the physical stability and imaging noise associated with this setup rendered the 12.5-cm depth impractical. With the thickest 6-cm standoff, the DFA often reached a depth of 12.5 cm but did so 40 mm from the bifurcation, a point more caudal and lateral to the femur, where the target would be so close to the bone that shadows/reflections would have blocked/reflected the therapy beam (again, this animal DBAC device had limited beam access compared to a multi-tile cuff wrapping around a limb). As a compromise, a target along the DFA approximately 20–25 mm from the bifurcation was chosen, making the deepest target depth 11.5 cm (with standoff layers included).

As noted earlier, two D&L cases did not meet bleeder characterization objectives (Table 2), both being slow bleeders (5 and 24 ml/min). Attempts to go to the extreme lower limit of the bleeder flow rate were influenced by the individual test subjects (e.g., alterations in both intra-aortic balloon inflation and stopcock manipulation), producing variability in the flow rate. In addition to potential variability of the low bleeder flow, it should be noted that the 4Z1c is a cardiac probe and thus likely had inadequate sensitivity in color-flow mode for this flow regime and would have benefited from additional power Doppler flow imaging mode (P-mode) optimization for the low-flow application. Also contributing to decreased flow sensitivity were the coupling and imaging noise challenges due to the TMM standoffs. Although the slow bleeder D&L is truly challenging, it is speculated that more in vivo practice experiments would have contributed to optimizations improving this performance.

While developing the blood flow control approaches, both the degree of intra-aortic balloon inflation and stopcock manipulation were evaluated in detail. Using the stopcock to regulate flow was found to have a demonstrable impact on the waveform signature of the flow, since blood pressure against the partial opening of the stopcock could generate non-bleed signatures. In contrast, using the balloon catheter to control the flow in the descending aorta produced a slow flow going through the superficial femoral artery with a valid “bleed” spectral Doppler signature, but it also quantitatively reduced the flow in the common femoral artery. It was realized that neither of the two techniques, individually, could provide both a realistic flow in the SFA and produce a valid spectral Doppler bleeding signature. As a consequence, the animal model was slightly modified such that the stopcock technique was used to generate a realistic flow rate in the proximal and distal femoral artery, as well as the SFAs, while the balloon catheter partial inflation technique was used to provide the bleed signatures during D&L spectral Doppler acquisition.

### Automated performance and device workflow

Although the DBAC automation objective was largely met with a “two-button” treatment being satisfied, one manual step (occurring between D&L and therapy) was required. This derived from the fact that two separate imaging systems had to be used—one for the D&L workflow and the other for the steps following initial D&L, including therapy. This was because full integration of the system software had not been completed by the date of the animal study evaluation. It was thus required that, after D&L, imaging probes from the first imager had to be manually switched to the other imager.

### Animal model acoustic complications

As described in reference [1], closed-loop localization of the HIFU beam could be done via acoustic radiation force impulse (ARFI) visualization of the beam focus using 3D rendering of the tissue strain pattern created by the focus in short test pulses. In this way, iterative feedback-based correction of the targeted beam could be done to align the beam with the vascular bleeder target. Although this method was demonstrated in a few of the in vivo treatments, the ARFI visualization was unable to reliably detect the foci of the therapy beams, in large part due to significant tissue motion induced by ventilator-driven respiration. It was found that the levels of tissue motion strain artifacts were on the same order as those induced by the HIFU test pulses themselves used to enable visualizing the focus. Thus, this problem was an artifact of the testing procedure, likely not representative of actual use of DBAC cuffs on limbs. In the future, however, motion compensation image processing will likely be necessary to properly segment and detect the ARFI-imaged targeting beam in the context of motion. In addition, D&L temporal image compounding, used to capture all arterial flow image data, was also affected by motion from ventilator-driven respiration, such as producing flash artifacts due to the extended acquisition times. Fortunately, a DBAC cuff device used on human extremities, being intimately pressed and clamped against the anatomy, should be more stable from a relative motion perspective when compared with the reported in vivo test.

Although the maximum thermal skin dose (MTSD) requirement was met, in 5 of 12 treatments, which were all shallow bleeders (≈50-mm deep), localized burns did occur on the actual animal skin. In these animals, the bleeder-branch target points were sometimes close to the skin (≈13–25 mm), thus requiring a TMM standoff (e.g., 3-cm thick). Unlike the case of an actual human DBAC treatment, this put the HIFU foci in proximity to the skin and the TMM-skin interface. In the skin burn cases, up to four small circular (*d* ≈ 5 mm) skin lesions occurred, usually in regular patterns matching thermal simulations, reflecting each active Tx array’s beam path through the skin. In this circumstance, some of the beams appeared to heat the TMM to a softening point and, when combined with the radiation force of the beam foci, created voids (5-mm diameter divots) in the TMM surface (visually confirmed post-experiment). The melting (divoting) of PVA standoffs seemed to occur more consistently when using 30-s doses (requiring higher intensities) in comparison to 60-s doses. Although only occurring in a small proportion of the treatments, the TMM divots are speculated to have contributed to the skin burns via acoustic reflections generated next to the skin. It is also possible that the voids reduced acoustic coupling and that the skin lesioning increased acoustic absorption at the skin surface. These behaviors, artifacts specific to this setup to achieve deep treatments, may have reduced energy delivery to the targets.

### DBAC efficacy

Achieving hemostasis, defined as coagulative occlusion of the target arteries, was the criterion for treatment success. Modest acute reductions in SFA blood flow were confirmed in one test subject. However, with the exception of the right SFA in animal #103 (complete occlusion, Fig. 13a) and left SFA in animal #104 (partial occlusion, Fig. 13b), there were no demonstrable differences between the baseline and post-therapy angiograms. Although angiography is a very sensitive indicator of vessel patency, it is less able to discern changes in the target vessels consistently seen on histopathology, which included evidence of blood clotting and collagen denaturation in all targeted SFAs. Interestingly, in the complete occlusion case, the SFA bleed flow rate reduced after the first dose but then appeared to slightly increase after the second dose, supporting the notion that occlusion occurred significantly after the dosing. None of the angiograms showed occlusion of the deep femoral artery, although DFA constriction was clearly evident in animal #104 based on angiography and necropsy observations.

### Acoustic streaming

The tourniquet function is needed to arrest net flow in the blood vessels, both to limit bleeding of the patient but also to minimize dissipation of the thermal energy and limit washout of coagulative components. Such dissipation is also possible from blood motion induced by the acoustic radiation force of the therapeutic beam itself (acoustic streaming).

Unlike a direct arterial puncture wound, which would produce blood leakage into the parenchymal tissue surrounding the vessel (constraining its movement), blood treated acoustically in a vessel is subject to acoustic streaming. This streaming “mixes” the blood, in effect, in an elongated tubular vessel cavity, with individual blood sub-volumes undergoing significant motion during the dose due to streaming. This behavior moves target blood in and out of the acoustic focal field and towards and away from portions of the vessel wall being heated. Further, enhanced convective cooling loss occurs as blood travels back and forth from warmer to cooler areas of the blood-filled vessel cavity. In the intact SFA, conditions do not constrain the blood to the treated region; thus, dwell time in the heated zone is decreased.

**Fig. 16:**
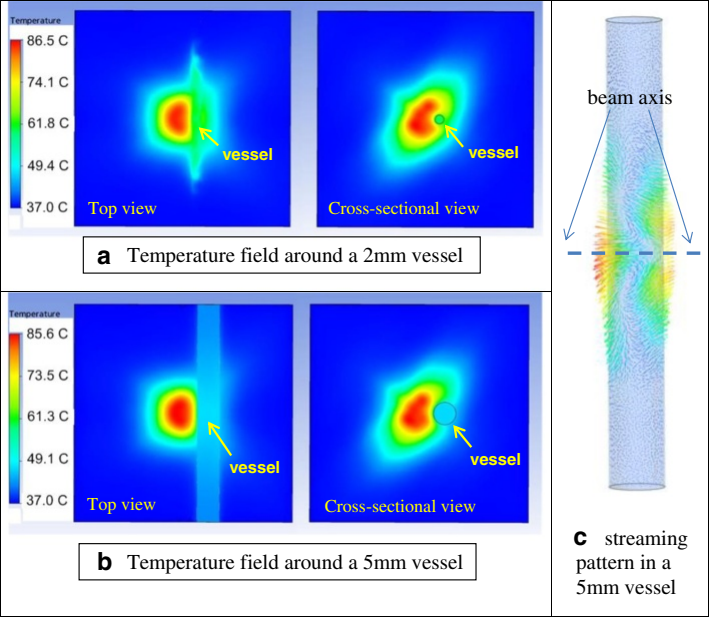
Temperature and acoustic streaming flow in the tissue medium with intact blood vessels of different size. Vessel is to the right of the central hot spot in each view. The fluid flow induced by acoustic streaming for the 5-mm vessel case (**b**) is shown in (**c**). The peak streaming velocity was 8.1 cm/s for the 2-mm vessel case (**a**) and 23.9 cm/s for the 5-mm vessel case (**b**)

FEM thermal and fluid dynamic simulations of streaming effects on tissue heating were performed by applying the radiation force as a momentum source to the blood pool in the vessel (similar to the methods of reference [5]), including applying a blood viscosity vs. temperature mechanism. Figure 16 shows a representative simulated temperature field in two orthogonal planes after a 30-s dose, with the focus being approximately 3 mm from the vessel wall. A 2-mm blood vessel is represented in Fig. 16a, and a 5-mm blood vessel in Fig. 16b (a typical porcine SFA in these studies was approximately 3 mm). With a baseline temperature of 37 °C, a maximum *T*_eod_ = 86 °C was achieved in tissue, while the blood in the 2-mm vessel had a *T*_eod_ = 66 °C (Fig. 16a), and merely a *T*_eod_ = 47 °C for the blood in the bigger vessel (Fig. 16b). Blood itself is a poor absorber of sound (*α* ≈ 0.2 dB/cm/MHz) and thus must derive most of its thermal exchange from contact (convective transfer) with tissue, so the vessel size has a significant impact on the flow pattern and thus the convective heat loss.

These streaming effects were experimentally confirmed (vs. simulations) in in vitro test phantoms and relate to the current in vivo results. Analysis of the partial occlusion treatment in animal #104, for example, showed that for the 60-s dose at 472 W/cm^2^ focal intensity (spatial average, temporal average), a peak acoustic streaming velocity of 6.1 cm/s is produced in the 3-mm SFA. This produced significant thermal dissipation in the blood, noting that the vessel-surrounding tissue had a *T*_eod_ = 91 °C, while the blood was only *T*_eod_ = 59.5 °C, on the low end of even fixed-temperature coagulation thresholds in the literature with these dwell times (60–70 °C; see Figure one in reference [1]). Also, even more thermal dissipation in the blood would likely occur in vivo (vs. the in vitro simulation results), since in vivo, the net radiation force was oriented at an acute angle to vessel axis, not orthogonal to it.

### Dose exposure time

Regarding coagulation efficacy, the required dose exposure limit of ≤30 s was inadequate for efficacy in this model, noting that the DBAC acoustic regime was predicated on “thermal coagulation” delivered by an autonomous device (for safety reasons it depends on controlled acoustic and heating behavior and avoiding unstable non-linear regimes). Also regarding control, due to high Tx driving voltages with 30-s doses, tile thermal management was more tractable for 60-s doses than 30-s doses (normalized to equivalent total dose energy), e.g., avoiding pre-set array thermal shutoff limits.

### Therapeutic acoustic regime

Although a thermal blood coagulation strategy was used in this study, both heat and mechanical stimuli can accelerate the coagulation cascade. Studies on the effect of ultrasound on platelets have demonstrated in vitro that therapeutic intensities can decrease the time to form a clot [6], and increase platelet aggregation, a key step in the coagulation cascade (likely through shear stress on surface membranes) [7]. Reinforcement of the hemostatic process may come from ultrasound enhancement of platelet activation [8], also attributable to acoustic shear forces and stimulation of platelets. It is noteworthy, however, that for significant platelet aggregation and activation increases to be produced by mechanical effects of ultrasound at acoustic frequencies relevant to DBAC, high focal intensities are required (e.g., *I* > 1000 W/cm^2^ at *f*_c_ = 1.1 MHz) [9]. Since such intensities at these frequencies are capable of producing cavitation, associated with the production of gas bubbles in the tissue, they come with potential complications regarding treatment control and safety.

It is speculated that non-thermal effects (the coagulation cascade) would likely have been suppressed in the SFA model due to the nature of the intact vessel. Since there was no vessel wall break, minimal direct contact of adventitial collagen with platelets occurred (which would have enhanced a normal coagulation cascade, triggering adhesion, leading to platelet activation, release of clotting factors, and progressing to platelet aggregation) [1]. Also relevant is the fact that an anti-coagulant (heparin) was required in this model.

Hemostasis using higher acoustic intensities has been demonstrated in bleeding vessels and organs [10–12]. In these earlier studies, the HIFU applicators were hand-held and moved during treatment, so it is difficult to interpret their time and spatially averaged intensities achieving hemostasis. These treatments involved surface, or near-surface, bleeding targets (less than 5-mm deep) with applicators that had water or metal coupling structures delivering highly localized power to the tissues (avoiding the attenuation losses that would apply to DBAC). In treating surgically exposed punctured blood vessels with intensities of 2500 and 3100 W/cm^2^ with a 3.5-MHz device, and using 500 and 2000 W/cm^2^ with a 2.0-MHz device (up to 3 min) [10], it was speculated that hemostasis was achieved through largely thermal effects, but the investigators indicated that it was unknown if cavitation was present. In performing hemostasis in lacerated liver [11] and spleen [12], high intensities (3000 W/cm^2^; pressures ≈ 4 MPa) were also used to achieve hemostasis but required relatively long average exposures (78 ± 44 and 96 ± 33 s, respectively). Interestingly, these organ hemostasis investigations did associate bleeding cessation with end-of-dose temperatures >70 °C (thermal mechanism) but reported the histology showed “homogenization” of the tissue, suggesting a strong mechanical effect. These studies used 9.6-MHz ultrasound, very absorptive for heating, but a frequency much too high for DBAC penetration needs.

Similar to findings in the current study, heating has long been known to occlude small blood vessels in surgery due to vessel wall constriction (from collagen shrinkage). Here the shrinkage force must overcome blood pressure, and the temperature required for collagen shrinkage increases with increasing load or tension (i.e., with blood pressure). The tensile strength of the collagen becomes stronger with higher temperature exposure [13] and, for a sustained temperature range 65 °C ≤ *T* ≤ 90 °C, showed maximum shrinkage (20–40 %) for exposures of 10 to 30 s [14], suitable for the DBAC application. Arterial hemostasis mediated by small artery collagen shrinkage has been shown to require temperatures in excess of 70 to 75 °C [13] for several seconds of heat exposure. Thus, small artery vessel shrinkage is a desirable supplemental mechanism to reinforce wound hemostasis.

## Summary and conclusion

A DBAC device was developed and tested in vivo, on porcine SFA (and DFA) bleeders. The applicator system was evaluated in a refereed in vivo final exam comprising 12 bleeder treatments (six SFA and six DFA targets) in three swine. All required elements of the testing were administered (except the maximum bleeder depth was 11.5 cm, not 12.5 cm). In vivo acoustic thermometry and ARFI-based closed-loop targeting were demonstrated, but unlike the case reported for the reference [1] system, they were not used to guide and monitor the in vivo treatments. Targeting, based on initial D&L coordinates alone without correction, showed 92 % of lesions centered on their vessel segment target areas. The overall image acquisition and D&L processing time for all tests was <5 min, well below the 10-min limit. Two D&L tests were unsuccessful with respect to bleeder characterization, both being “low flow” bleeders and erroneously indicated as non-bleeder vessel bifurcations.

The primary automation objective was achieved, with only a few “manual” steps being inserted during the testing. Specifically, the “two-button” treatment was satisfied but required manual switching (between imaging systems) of the Ix probe connectors, and manual targeting markers were placed for the DFA bleeders (since they were not catheterized).

For the primary objective of hemostasis, the required dose exposure limit of 30 s was found inadequate for this treatment regime, so 60-s dosing and dual-dosing was explored. Acute reductions in blood flow (20–30 %) were achieved in one subject, but only two SFA vessel occlusions (one partial, one complete) were confirmed angiographically. The successful complete coagulative occlusion was achieved with a double dose (60 + 30 s = 90 s total) using a spatial- and time-averaged focal intensity approaching *I* ≈ 500 W/cm^2^. Although angiography indicated that hemostasis may have been partly due to vessel wall constriction (collagen shrinkage), gross and microscopic histopathology showed a thrombogenic effect on targeted arteries, contributing to accumulating vascular embolization. Encouragingly, the collateral damage assessment indicated the treatments to be localized and accurately targeted, with most treatments showing minimal histologic effects on surrounding structures, including the parent artery (CFA).

The SFA/DFA porcine model was the most suitable animal model to meet specified in vivo parameter ranges. In addition, the model was stable throughout the long (multi-hour) test protocols. The animal model was, however, imperfect in simulating DBAC cuff treatment on human limbs, especially regarding the means needed to achieve bleeder depth and flow rate control, and the device-tissue relative motion induced by ventilator-supported breathing. In particular, the TMM standoffs used to address the need for greater target depths came at a cost of impacting acoustic performance and adding logistical challenges. The use of an intact vessel as the surrogate bleeder was a necessary compromise, in that a direct-puncture wound would have rendered too difficult the quantification and control of blood flow rates. Had a direct-puncture arterial wound been possible (especially without heparinization), a more realistic and biologically accurate model may have resulted (e.g., coagulation cascade activity), one also amenable to more effective blood heating (e.g., due to disruption of acoustic streaming). While significant accomplishments were made with the SFA/DFA approach, the model constraints suggest that an improved animal model be further pursued in future in vivo bleeder testing.

If not meeting all in vivo objectives, the performance of the animal DBAC applicator results were positive. In particular, as supported by the automated sequence execution, the D&L results showed accurately targeted multi-array power delivery, and based upon the evidence of collagen denaturation and thrombogenesis in the targeted vessels, the performance of this research prototype system was encouraging.

## Abbreviations

ACUSON SC2000: Siemens commercial ultrasound imaging system
ARFI: acoustic radiation force impulse imaging
B-mode: ultrasound gray scale imaging
CAD: computer-assisted design
CIMU: Center for Industrial and Medical Ultrasound (at University of Washington)
CFA: common femoral artery
DARPA: Defense Advanced Research Projects Agency
D&L: detection and localization; function/subsystem for finding bleeder sites
C7F2: a 2D imaging probe (used to supplement and backup the 4Z1c)
DBAC: Deep Bleeder Acoustic Coagulation
DFA: deep femoral artery—the vessel branch used for deeper bleeder targets; a continuation segment of the femoral artery beyond SFA bifurcation
*f*_c_: acoustic center frequency (in MHz)
FEM: finite element method (computational modeling method)
HIFU: high-intensity focused ultrasound
*I*: acoustic intensity (in W/cm^2^)
Ix: imaging probe/transducer or imaging probe functions
MTSD: maximum thermal skin dose: *T*_eod_ ≤ 52 °C, or ΔTskin_eod_ ≤ 20 °C
P-mode: power Doppler flow imaging mode
PVA: polyvinyl alcohol (base material for TMM used in standoffs)
RI: Resistive Index
RNN: recurrent neural network; method used in acoustic thermometry for this project
SD: Spectral Doppler (ultrasound flow velocity measurement method)
SFA: superficial femoral artery (vessel branch defined as a bleeder)
*T*: temperature (in °C)
*T*_eod_: end-of-dose temperature
TIPS: Texas A&M Institute for Preclinical Studies
TMM: tissue-mimicking material—used for surrogate tissue standoffs
Tx: therapeutic array or therapeutic array functions
α: coefficient of acoustic attenuation/absorption (in dB/cm/MHz)
Δ*T*_eod_: end-of-dose temperature rise relative to baseline temperature starting point
4Z1c: ultrasound 3D volume imaging probe (connected to ACUSON SC2000 imager)

## Footnotes

1 The DBAC device design and objectives were guided by the requirements and milestones agreed to with regard to the Phase II DBAC Cuff Contract, DARPA HR0011-08-3-0004.

2 The Texas Institute for Preclinical Studies (TIPS), Texas A&M University, provided the test facility and administered the *in vivo* test bed tests, with assistance from University of Washington test monitors. The DBAC animal model was developed jointly at TIPS and University of Washington Center for Industrial and Medical Ultrasound (CIMU).
